# Characterization of sensory and motor dysfunction and morphological alterations in late stages of type 2 diabetic mice

**DOI:** 10.3389/fendo.2024.1374689

**Published:** 2024-03-11

**Authors:** Ting Tian, Haofeng Li, Sensen Zhang, Maojun Yang

**Affiliations:** ^1^ School of Rehabilitation Sciences and Engineering, University of Health and Rehabilitation Sciences, Qingdao, China; ^2^ Ministry of Education Key Laboratory of Protein Science, Beijing Advanced Innovation Center for Structural Biology, Beijing Frontier Research Center for Biological Structure, School of Life Sciences, Tsinghua University, Beijing, China; ^3^ Cryo-EM Facility Center, Southern University of Science and Technology, Shenzhen, China

**Keywords:** diabetic neuropathy, behavioral assessment, sensory loss, disturbed locomotion, morphological alteration, muscle atrophy, neuromuscular transmission, synaptic inputs

## Abstract

Diabetic neuropathy is the most common complication of diabetes and lacks effective treatments. Although sensory dysfunction during the early stages of diabetes has been extensively studied in various animal models, the functional and morphological alterations in sensory and motor systems during late stages of diabetes remain largely unexplored. In the current work, we examined the influence of diabetes on sensory and motor function as well as morphological changes in late stages of diabetes. The obese diabetic Lepr^db/db^ mice (db/db) were used for behavioral assessments and subsequent morphological examinations. The db/db mice exhibited severe sensory and motor behavioral defects at the age of 32 weeks, including significantly higher mechanical withdrawal threshold and thermal latency of hindpaws compared with age-matched nondiabetic control animals. The impaired response to noxious stimuli was mainly associated with the remarkable loss of epidermal sensory fibers, particularly CGRP-positive nociceptive fibers. Unexpectedly, the area of CGRP-positive terminals in the spinal dorsal horn was dramatically increased in diabetic mice, which was presumably associated with microglial activation. In addition, the db/db mice showed significantly more foot slips and took longer time during the beam-walking examination compared with controls. Meanwhile, the running duration in the rotarod test was markedly reduced in db/db mice. The observed sensorimotor deficits and motor dysfunction were largely attributed to abnormal sensory feedback and muscle atrophy as well as attenuated neuromuscular transmission in aged diabetic mice. Morphological analysis of neuromuscular junctions (NMJs) demonstrated partial denervation of NMJs and obvious fragmentation of acetylcholine receptors (AChRs). Intrafusal muscle atrophy and abnormal muscle spindle innervation were also detected in db/db mice. Additionally, the number of VGLUT1-positive excitatory boutons on motor neurons was profoundly increased in aged diabetic mice as compared to controls. Nevertheless, inhibitory synaptic inputs onto motor neurons were similar between the two groups. This excitation-inhibition imbalance in synaptic transmission might be implicated in the disturbed locomotion. Collectively, these results suggest that severe sensory and motor deficits are present in late stages of diabetes. This study contributes to our understanding of mechanisms underlying neurological dysfunction during diabetes progression and helps to identify novel therapeutic interventions for patients with diabetic neuropathy.

## Graphical Abstract

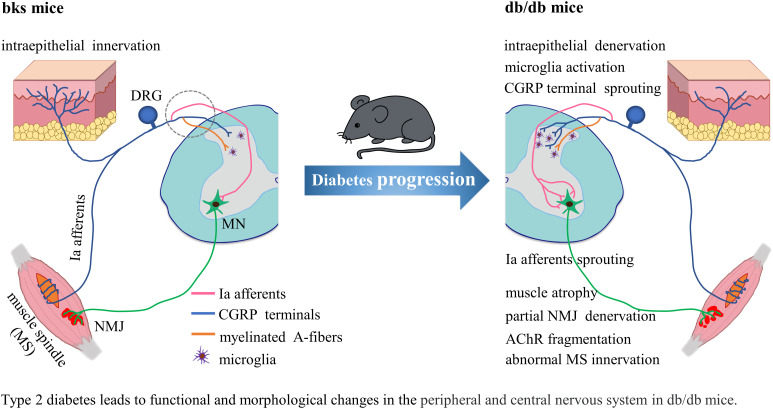

## 1 Introduction

Diabetic neuropathy is a neurodegenerative disorder that mainly affects peripheral and autonomic nervous systems via damaging sensory, autonomic, and motor axons (1). Diabetic neuropathy is the most prevalent syndrome among diabetes-related complications and affects approximately half of individuals with type 1 or type 2 diabetes (2). Diabetic neuropathy leads to distal-to-proximal symmetric polyneuropathy, which is characterized by the “stocking and glove” pattern of symptoms (2), and heavily affects quality of life by losing sensory function, increasing falls and neuropathic pain, resulting in substantial physical disability and morbidity (1, 3, 4). The pathophysiology of diabetic neuropathy is multifaceted and complicated. Glycaemic control shows certain therapeutic benefit on diabetic neuropathy in type 1 diabetes, while has modest effect in patients with type 2 diabetes (2). Accordingly, diabetic neuropathy prevention is challenging and lacks effective treatments.

Substantial studies have documented sensory and autonomic dysfunction in murine models of diabetes. In the streptozotozin (STZ)-induced type 1 diabetic mice, reduced sensitivity to mechanical and thermal stimuli and decreased motor nerve conduction velocity were observed (5). Nevertheless, robust mechanical allodynia was developed in some STZ-induced mouse model (6). Similarly, the type 2 diabetic rats induced by STZ and high-fat diet displayed mechanical and thermal hyperalgesia within 12 weeks after STZ-injection, and also developed nerve demyelination and loss of intraepidermal nerve fibers (7). Impaired mechanosensation and thermal nociception were also observed in the diabetic Ins2+/Akita mouse (8). In the db/db mouse model of type 2 diabetes, the density of blood vessels and intraepidermal nerve fibers was decreased during the age of 20 to 24 weeks (9), and the thermal and mechanical sensitivity were downregulated compared with control animals from the age of 18 to 28 weeks (10, 11). Additionally, the db/db mice developed severe autonomic neuropathy at early time points, and significantly reduced autonomic innervation of sweat glands and the rate of sweat droplet formation in the footpads (12). However, motor neuropathy develops relatively late during diabetes progression, and diabetes-induced motor phenotypes and histological alterations have not been extensively investigated in preclinical models.

In the present study, we examined the functional and morphological changes of sensory and motor system in late stages of type 2 diabetic mice. We assessed sensory and motor function in diabetic mice by several behavioral assessments, including mechanical stimulus, hot plate, beam-walking test and rotarod test. We also detected neuromuscular transmission efficacy with electromyography and analyzed structural alterations in the peripheral and central nervous system using H&E and immunostaining. We confirmed severe behavioral deficits and underlying morphological alterations in diabetic mice. Severe loss of epidermal nerve fibers, evident muscle atrophy, attenuated neuromuscular transmission, and imbalanced excitatory-inhibitory synaptic inputs were observed in diabetic mice and are presumed to contribute to the pronounced sensory and motor abnormalities. These results will extend our understanding in the development and progression of neurological dysfunction, and contribute to identify mechanisms underlying neurological abnormalities and provide potential therapeutic targets for diabetic neuropathy.

## 2 Methods

### 2.1 Animals

The BKS. Cg-m+/+Lepr^db^/J (db/db) diabetic mice were purchased at 6 weeks of age from Laboratory Animal Resources Center, Tsinghua University. Age-matched C57BLKS/J (bks) mice were used as nondiabetic control animals. Mice were housed in groups of 4 on a 12-hour light/dark cycle in a humidity- and temperature- controlled room with free access to food and water throughout the study. Blood glucose and body weight were measured every six weeks after animals reached 8 weeks of age. Diabetic mice aged 32 weeks and age-matched bks mice were used in behavioral tests and electromyography assessments. The mice were sacrificed for further morphological analyses at the end of the experiment. All experimental procedures performed on animals were approved by the Institutional Animal Care and Use Committee (IACUC) of Laboratory Animal Resources Center, Tsinghua University (approval No. 864) on December 9, 2022.

### 2.2 Blood glucose and body weight

Blood glucose concentration and body weight were measured every six weeks at the same time in the afternoon from 8 weeks of age until 32 weeks of age. Blood samples were acquired from the tail vein of each mouse after tail disinfection with alcohol-soaked cotton and then tested using a glucose meter (Yuwell 660). The tail was pressed for 10 seconds to stop bleeding after blood collection. Body weight was recorded after blood glucose measurement.

### 2.3 Behavioral tests

For behavioral tests, animals were habituated to each experimental apparatus for 60 min/d for three consecutive days before the test. The observers were blinded to the grouping. No data were excluded from statistical analyses.

### 2.4 Thermal sensitivity

To test thermal sensitivity, mice were placed in a transparent plastic cylinder on a metal plate (BIO-CHP) preheated to 52°C, and the latency to jumping, hindpaw licking, or shaking was recorded. A cut off of latency of 30 s was set to avoid tissue burning (13). Three consecutive recordings were performed per animal at 15-minute intervals.

### 2.5 Mechanical sensitivity

Mechanical sensitivity was measured using a dynamic plantar aesthesiometer (Ugo Basile, 37550). In brief, mice were placed in plastic chambers on an elevated steel mesh, and then the movable touch stimulator was applied with pressure to the plantar surface of the hindpaw to measure the mechanical withdrawal threshold. Three trials per animal were performed at 15-min intervals.

### 2.6 Rotarod test

Locomotor ability, balance, and coordination in mice were assessed using a rotarod apparatus (Ugo Basile, 47650). The mice had rotarod training for three days prior to testing. For the training period, the mice were placed on the rotarod apparatus at 5 rpm for 5 min. On the fourth day, mice were placed on an accelerating rotarod cylinder that gradually increased from 5 to 20 rpm within 5 min, and the latency to fall was recorded. Three trials per animal were repeated.

### 2.7 Beam-walking test

A modified wooden beam apparatus (100 cm length, 1 cm width) was suspended 50 cm above the floor, with the starting end illuminated by a desk lamp and the other end attached to an enclosed box. Mice were pretrained three times per day for three days. For the training period, mice were placed at the illuminated end of the beam and were trained to cross the beam within one minute. On the fourth day, the time to walk across the beam and the number of hind foot slips were recorded. At least three trials were repeated per animal.

### 2.8 Electromyography recordings

Compound muscle action potentials (CMAPs) in the tibialis anterior (TA) were measured to evaluate the conduction efficiency of the tibial nerve. Mice were anesthetized with isoflurane (2%-3% induction and 1.5% maintenance in oxygen) and placed on a heating blanket to maintain body temperature throughout the test and until waking. The bipolar hooked stimulating electrode was positioned under the exposed left sciatic nerve, the recording electrode was implanted in the TA muscle, and the reference electrode was attached to the plantar skin of the hindlimb. The amplitude of CMAP was measured using a multi-channel physiological signal acquisition and processing system (RM6240XC).

### 2.9 Tissue processing

Animals were anesthetized with isoflurane (2-3% in oxygen) and then transcardially perfused with 0.9% normal saline solution, followed by 4% paraformaldehyde (PFA) in phosphate buffer. The spinal cords, sciatic nerves, TA muscles, gastrocnemius (GM) muscles, paw skin, and pancreas were dissected, and post-fixed in 4% PFA at 4°C for 12 h, and then dehydrated in 30% sucrose solution for 48 h. For muscle wet weight and muscle fiber area analysis, the fresh TA and GM muscles were collected and immediately weighed, and then were snap-frozen in isopentane solution pre-cooled with liquid nitrogen. The prepared tissues were then embedded in optimal cutting temperature compound (OCT) and sectioned using a cryostat (Leica, CM1950). Tissue sections were stored at -20°C until further histological assessment.

### 2.10 Hematoxylin and eosin staining

H&E staining was performed to evaluate muscle atrophy. In brief, the muscle tissue sections were rinsed with distilled water and stained with hematoxylin for 10 min, then rinsed with distilled water for 2 min and differentiated with 1% hydrochloric alcohol solution for 15 s. After that, sections were immersed in bluing reagent for 10 s and washed with distilled water. Next, the sections were stained with eosin for 2 min and soaked in gradient alcohol and xylene sequentially, and then sealed using neutral balsam. Finally, images of the sections were captured with an Olympus BX63 fluorescence microscope, and images were analyzed using ImageJ.

### 2.11 Immunostaining

The frozen tissue sections were washed in phosphate buffer saline (PBS) and permeabilized with 0.3% Triton X-100 for 1 h at room temperature. After blocking with 10% normal goat serum for 1 h at room temperature, the sections were incubated with primary antibodies at 4°C for 48 h. The primary antibodies used included: anti-insulin, rabbit,1:500, Abcam, Cat# ab181547, RRID: AB_2716761; anti-PGP9.5, rabbit, 1:200, Abcam, Cat# ab108986, RRID: AB_10891773; anti- CGRP, rabbit, 1:1000, CST, Cat# 14959S, RRID: AB_2798662; anti-Neurofilament heavy polypeptide (NEFH), rabbit, 1:400, Abcam, Cat# ab207176, RRID : AB_2827968; anti-Iba1, rabbit, 1:1000, Abcam, Cat# ab178846, RRID: AB_2636859; anti-IB4, 1:400, Sigma, Cat# L2140, RRID: AB_2313663; anti-Synaptophysin, 1:200, rabbit, Abcam, Cat# ab32127, RRID: AB_2286949; anti-NeuN, rabbit, 1:500, Abcam, Cat# ab177487, RRID: AB_2532109; anti-NeuN, mouse, 1:1000, Abcam, Cat# ab104224, RRID: AB_10711040; Anti-VGLUT 1, Guinea Pig, 1:1000, Synaptic Systems, Cat# 135 304, RRID: AB_887878; Anti-VGLUT2, rabbit, 1:400, Abcam Cat# ab216463, RRID: AB_2893024; Anti-VGAT, mouse, 1:400, Abcam, Cat# ab211534, RRID: AB_292422. After three washes with PBS, the sections were incubated with secondary antibodies for 2 h at room temperature. The secondary antibodies used included: Alexa Fluor™ 488 donkey anti-Mouse IgG, Thermo Fisher Scientific, Cat# A-21202, RRID : AB_141607; Alexa Fluor™ 594 donkey anti-Mouse IgG, Thermo Fisher Scientific, Cat# A-21203, RRID : AB_141633; Alexa Fluor 594 donkey anti-Rabbit IgG, Thermo Fisher Scientific, Cat# A-21207, RRID : AB_141637; Alexa Fluor™ 488 donkey anti-Rabbit IgG, Thermo Fisher Scientific, Cat# A-21206, RRID : AB_2535792; Alexa Fluor 633 goat anti-Guinea Pig IgG, Thermo Fisher Scientific, Cat# A-21105, RRID : AB_2535757; DyLight™ 594 streptavidin Protein, Thermo Fisher Scientific, Cat# 21842, RRID : AB_2619631. The sections were washed with PBS and labeled with Hoechst for nuclear staining. After final washes with PBS, the sections were mounted with antifade solution. Images were acquired using a spinning-disk confocal microscope (Andor Dragonfly), and images were processed and quantified with ImageJ or Imaris 9.9.1.

### 2.12 Statistical analysis

All data are presented as mean ± SEM, and data processing and statistical analyses were performed using GraphPad Prism 9.0. Comparisons between two groups were performed using unpaired Student’s *t* test when data were normally distributed, and the non-parametric Mann-Whitney test otherwise. The blood glucose and body weight data were analyzed by two-way ANOVA with *post hoc* Bonferroni test. Significance was determined as *P < 0.05, ** *P* < 0.01, *** *P* < 0.001, **** *P* < 0.0001.

## 3 Results

### 3.1 Sensory and motor deficits in late stages of diabetic mice

**Figure 1:**
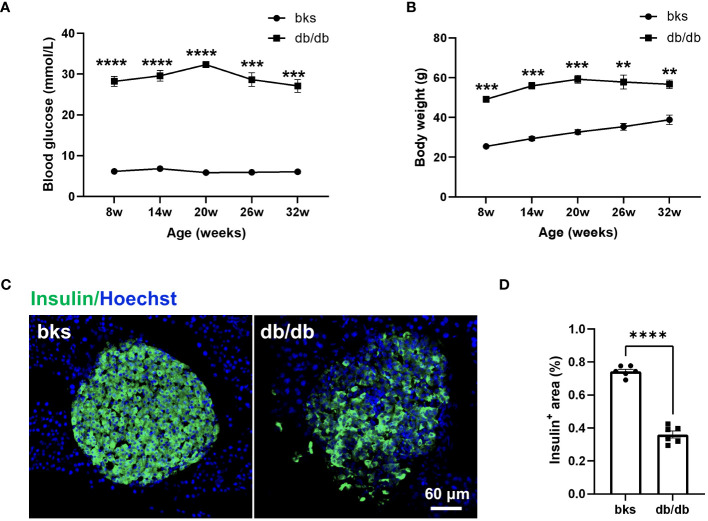
Diabetic db/db mice exhibit hyperglycemia, obesity, and loss of insulin-producing β-cells throughout disease progression. **(A, B)** Blood glucose concentrations **(A)** and body weight **(B)** in diabetic db/db and bks control mice at indicated time points. **(C)** Representative immunostaining for insulin-producing β-cells in islets of pancreas tissue from diabetic and control mice aged 32 weeks. **(D)** Quantification of insulin-positive area in **(C)**, the insulin-positive area is normalized to the area of the islets. Data are presented as mean ± SEM, n = 6 mice in each group, **P < 0.01, ***P < 0.001, ****P < 0.0001, two-way ANOVA with *post hoc* Bonferroni test in **(A, B)**, and unpaired t-test in **(D)**.

The db/db mouse model is commonly used to study diabetes, but experiments are typically conducted in relatively young mice. To confirm the model is appropriate to study sensory and motor function as well as pathology in later stages of diabetes, we first characterized blood glucose and weight in db/db and control bks mice from 8 through 32 weeks of age. The db/db mice exhibited higher blood glucose levels compared with controls at the first testing (8 weeks of age) and developed severe hyperglycemic symptoms that were sustained until the last time point tested (32 weeks of age) (**Figure 1A**). The diabetic mice also gained significantly more weight than control mice during the observation period (**Figure 1B**). In addition, the persistent hyperglycemia leads to destruction of functional insulin-producing β-cells, immunostaining analysis of the harvested pancreas tissue showed that the percentage of insulin-positive area was profoundly decreased in db/db mice compared with control mice (**Figures 1C, D**).

**Figure 2:**
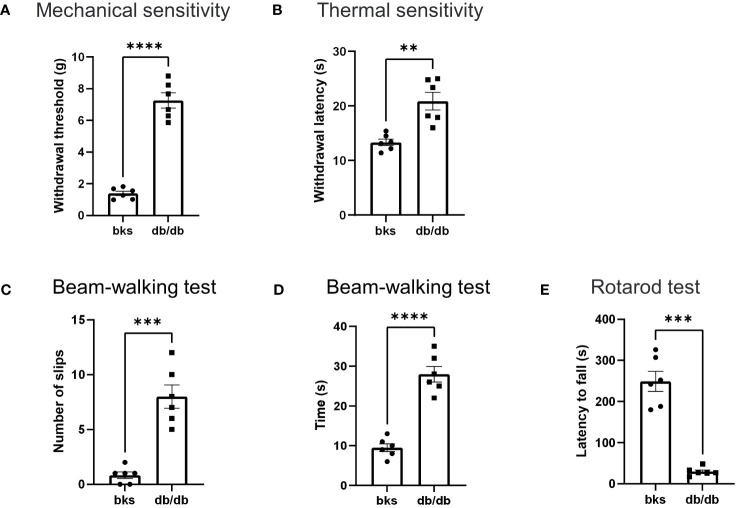
Behavioral assessments in late stages of type 2 diabetic mice. **(A, B)** Mechanical threshold **(A)** and withdrawal latency **(B)** of hindpaws in db/db and bks mice. **(C)** Number of foot slips in diabetic and control mice in the beam-walking test. **(D)** Time taken to walk cross the beam in two groups. **(E)** The latency to fall from the spinning rotarod in diabetic and control mice. Data are presented as mean ± SEM, n = 6 mice in each group, **P <0.01, ***P < 0.001, ****P < 0.0001, unpaired t-test.

Loss of sensation is the primary feature of diabetic neuropathy, which occurs early in diabetes progression. Therefore, we first tested mechanical and thermal sensitivity in diabetic mice using the dynamic plantar aesthesiometer and hot plate. Similar to previous studies in db/db mice at age 20 to 28 weeks (14), the diabetic mice in our study developed typically diabetic sensory neuropathy. The db/db mice had markedly higher withdrawal threshold of hindpaws compared with age-matched control mice (**Figure 2A**). Likewise, the thermal latency was also upregulated in db/db mice in comparison to controls (**Figure 2B**). These results indicated that sensory hyposensitivity is pronounced during the late stages of diabetes.

Next, we investigated whether db/db mice developed motor deficits during the late-stage diabetes with beam-walking test to assess the sensorimotor ability and motor coordination (15, 16). The db/db mice had significantly more foot slips during the beam-walking test in comparison to controls (**Figure 2C**). Moreover, the time taken to cross the beam apparatus was notably longer in db/db mice compared with control group (**Figure 2D**). These results suggest that db/db mice exhibited deficits in sensorimotor function involving balance and gait performance. Furthermore, we assessed locomotion ability using the rotarod test, and we observed a significant difference in rotarod performance between the diabetic and control groups. The running duration on the spinning rotarod was profoundly reduced in db/db mice compared to controls (**Figure 2E**), suggesting that db/db mice displayed severe impairment in locomotor ability. Taken together, these data demonstrated that type 2 diabetes induces significant sensorimotor and locomotion dysfunction in addition to sensory abnormalities during late disease progression in db/db mice.

### 3.2 Degeneration of intraepithelial nerve fibers in the footpad of db/db mice

**Figure 3:**
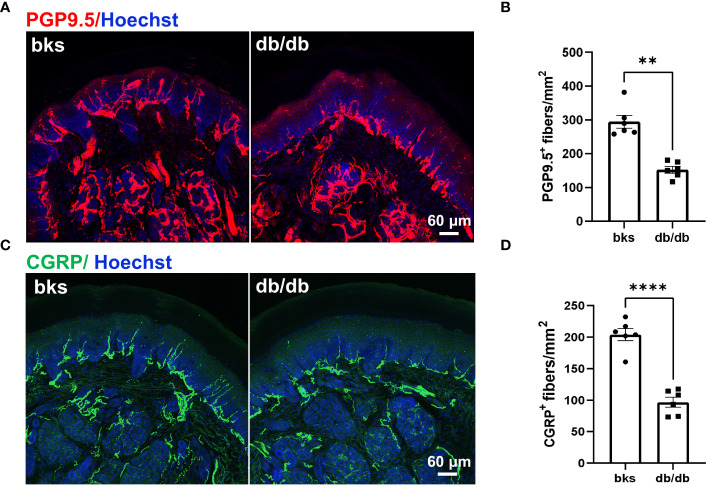
Sensory innervation of footpad epidermis in diabetic mice at the age of 32 weeks. **(A)** Representative images of PGP9.5 staining for IENFs in diabetic and control mice. **(B)** Mean density of IENFs in footpad epidermis in diabetic and control mice. **(C)** Representative CGRP staining of footpad epidermis in diabetic and control mice. **(D)** Mean density of CGRP-positive fibers in footpad epidermis of diabetic and control mice. Data are presented as mean ± SEM, n = 6 mice in each group, **P < 0.01, ****P < 0.0001, non-parametric Mann-Whitney test in **(B)** and unpaired t-test in **(D)**. IENFs, intraepithelial nerve fibers.

Reduced sensation is the primary symptom in patients with diabetic neuropathy, and this is mainly due to loss of intraepithelial nerve fibers (IENFs) during diabetes progression (10). Therefore, we detected the epidermal innervation of footpad skin using immunostaining in these diabetic mice. We first labeled the IENFs by immunostaining for PGP9.5. The density of PGP9.5-positive fibers in footpad epidermis was markedly reduced in db/db mice compared with that in the controls (**Figures 3A, B**), indicating degeneration of IENFs in aged diabetic mice. The decreased sensitivity to noxious mechanical and heat stimuli (**Figures 2A, B**) might be attributed to the retraction of intraepithelial nociceptive fibers in the plantar skin. We then marked nociceptive peptidergic fibers by CGRP immunostaining and found that the density of the CGRP-immunoreactive fibers was also significantly decreased in db/db mice (**Figures 3C, D**), suggesting extensive nociceptive fiber denervation in footpad epidermis during later-stage diabetes.

### 3.3 Sprouting of CGRP-positive terminals in the spinal dorsal horn of db/db mice

The primary afferent neurons within the dorsal root ganglia (DRG) detect the internal and external stimuli by peripheral terminals, and transmit the information to the spinal dorsal horn (SDH) for sensory processing via central terminals (17, 18). Any disruption of these neural circuits leads to sensory abnormalities. The impaired sensory perception and behavioral sensitivity we observed in db/db mice (**Figures 2A, B**) was mainly associated with the remarkable loss of IENF, particularly nociceptive fibers (**Figure 3**). We then determined whether the central terminals of these primary afferent neurons in the SDH were also involved in the decreased noxious sensitivity.

**Figure 4:**
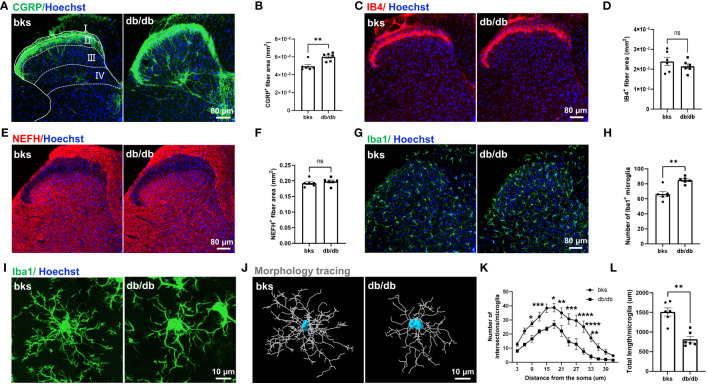
Central terminal distribution of primary afferent sensory neurons in the SDH of L4-L6 spinal segments from diabetic mice aged 32 weeks. **(A)** Representative images of CGRP-positive terminals in the SDH of diabetic and control mice, the laminae of the SDH are denoted by white dotted lines. **(B)** Quantification of CGRP-positive fiber area in **(A)**. **(C)** Representative images of IB4-positive terminals in the SDH from diabetic and control mice. **(D)** Quantification of IB4-positive fiber area in **(C)**. **(E)** Representative images of NEFH-positive terminals in the SDH of diabetic and control mice. **(F)** Quantification of NEFH-positive fiber area in **(E)**. **(G)** Representative images of Iba1-positive microglia in the SDH of diabetic and control mice. **(H)** Quantification of microglia in **(G)**. **(I)** High magnification of Iba1 staining showing ramified microglia transitioned into reactive microglia in db/db mice. **(J)** Morphology tracing of microglia stained with Iba1 in **(I)**. **(K)** Sholl analysis of branching complexity of microglia in db/db and control mice. **(L)** Total length of microglia branches in two groups. Data are presented as mean ± SEM, n = 6 mice in each group, *P < 0.05, **P < 0.01, ***P < 0.001, ****P < 0.0001, ns, no significant difference, non-parametric Mann-Whitney test in **(B, H, L)**, unpaired t-test in **(D, F)** and ordinary two-way ANOVA in **(K)**. SDH, spinal cord dorsal horn, NEFH, neurofilament heavy polypeptide.

We evaluated the morphological distribution of CGRP-positive nociceptive peptidergic C-fibers in the SDH from the L4-L6 spinal segments. Consistent with previous studies (19, 20), the CGRP-positive terminals were primarily located in lamina I and outer lamina II of the SDH in nondiabetic control mice. Unexpectedly, there was no degeneration of CGRP-positive terminals in db/db mice, on the contrary, the CGRP-positive terminals were extensively distributed in both superficial lamina (I–II) and deep laminae (III–IV) in the SDH. The area of CGRP-positive terminals was markedly elevated in db/db mice as compared to the controls (**Figures 4A, B**), suggesting sprouting of CGRP-positive terminals during diabetes progression. We also detected the distribution of nonpeptidergic C-fibers and myelinated A-fibers in the SDH by IB4 and NEFH labeling, respectively. In contrast, no difference was found in the area of IB4-positive and NEFH-positive terminals between diabetic and nondiabetic mice (**Figures 4C–F**).

A previous study indicated that CGRP-positive terminal sprouting in the SDH was associated with hyperactivation of microglia (21). To explore the mechanisms underlying sprouting of CGRP-positive terminals late during diabetes progression, we performed immunostaining for Iba1 (a marker of microglia) in the SDH and found that the number of Iba1-positive microglia was increased in db/db mice compared with controls (**Figures 4G, H**). In control mice, the majority of microglia were in a resting state and characterized by numerous thin ramifications, whereas, in db/db mice, the morphology of microglia was transitioned from highly ramified processes to retracted processes (**Figures 4I, J**). The number of branch intersections and total branch length of microglia were significantly decreased in db/db mice compared to control group (**Figures 4K, L**), suggesting a reactive state of microglia. These results indicated that microglia activation was induced in late-stage diabetes and might contribute to the sprouting of CGRP-positive terminals in the SDH.

### 3.4 Muscle atrophy and abnormal neuromuscular transmission were involved in motor dysfunction in db/db mice

**Figure 5:**
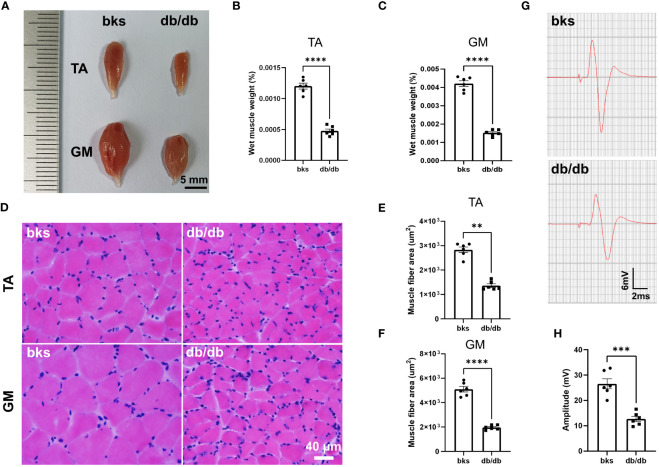
Diabetic mice displayed severe muscle atrophy and declined neuromuscular transmission at 32 weeks of age. **(A)** Gross morphology of TA and GM muscles in diabetic and control mice. **(B, C)** Wet weight of TA **(B)** and GM **(C)** muscles in diabetic and control mice. The muscle weight was normalized to body weight (n = 6). **(D)** HE staining of TA and GM muscle fibers in diabetic and control mice. **(E, F)** Quantification of mean CSA of TA **(E)** and GM **(F)** muscle fibers in diabetic and control mice (n = 6). **(G)** Representative images of CMAPs in diabetic and control mice. **(H)** Quantification of CMAP amplitude in **(G)** (n = 6). Data are presented as mean ± SEM, **P < 0.01, ***P < 0.001, ****P < 0.0001, unpaired t-test in **(B, C, F, H)** and Mann-Whitney test in **(E)**. TA, tibialis anterior; GM, gastrocnemius; CSA, cross sectional area; CMAPs, compound muscle action potentials.

The db/db mic displayed severe deficits in locomotor ability as observed in beam-walking and rotarod tests (**Figures 2C–E**). We next investigated the mechanisms underlying motor dysfunction. Skeletal muscles are the final executor of locomotion, and rhythmic muscle contractions ensure coordinated movement (22, 23). Skeletal muscle atrophy leads to myasthenia and motor abnormalities in pathological conditions (24). We therefore determined whether muscle atrophy was present in db/db mice by assessing the gross morphology and wet weight of TA and GM muscles. For gross morphology, the TA and GM muscles in nondiabetic mice were thick and plump, while the muscles became smaller and thinner in db/db mice as shown in **Figure 5A**. Consistently, the wet weight of TA and GM muscles was significantly reduced in db/db mice compared with controls (**Figures 5B, C**). Cross sectional area (CSA) of muscle fiber was further calculated to evaluate muscle atrophy. H&E staining showed that the CSA of TA and GM muscle fiber was evidently reduced in db/db mice in comparison to control mice (**Figures 5D–F**). Accordingly, these data indicated that the db/db mice developed severe muscle atrophy in lower extremities in late diabetes progression.

A variety of conditions can cause muscle atrophy, including malnutrition, aging, muscle disuse or denervation (24). Denervation of neuromuscular system or malfunctioning of neuromuscular transmission results in loss of muscle mass and function (25), and we hypothesized these abnormalities may be involved in db/db mice based on the above motor dysfunction. We therefore performed evoked electromyography to test the electrophysiologic properties of neuromuscular transmission, and recorded CMAPs to evaluate the nerve-induced action potential activity in TA muscle. The CMAP amplitude in TA muscle was reduced by 55.68% in db/db mice compared to controls (**Figures 5G, H**), suggesting abnormal neuromuscular conduction strength in late stage of type 2 diabetic mice.

### 3.5 The db/db mice displayed abnormal innervation of neuromuscular junction and muscle spindle

**Figure 6:**
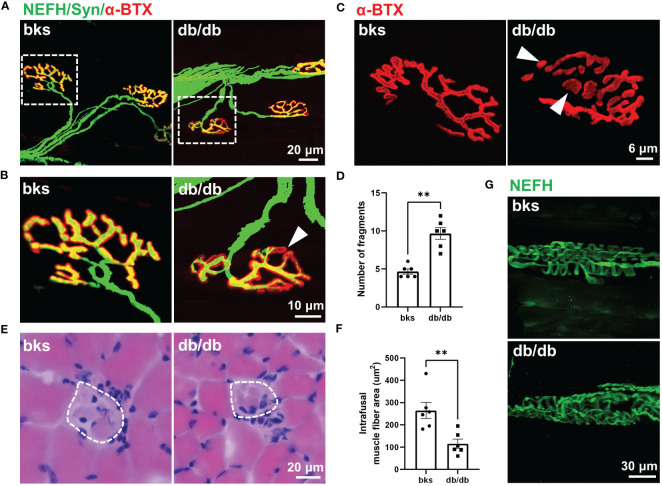
Abnormal innervation of NMJs and muscle spindle in aged diabetic mice. **(A)** An overall view of the NMJs in TA muscle from diabetic and control mice. **(B)** High-magnification images of NMJ in the dotted boxed region in **(A)**. The arrowhead indicates partial denervation of the NMJ. **(C)** Representative images of AChR clusters with α-BTX staining in diabetic and control mice. Arrowheads indicate AChR cluster fragments. **(D)** Quantification of AChR cluster fragments in **(C)**. **(E)** H&E staining of the equatorial region of TA muscle spindles in diabetic and control mice. The dashed line indicates the spindle capsule that encircles the intrafusal muscle fibers. **(F)** Quantification of mean CSA of intrafusal muscle fiber in **(E)**. **(G)** Representative immunostaining images of TA muscle spindles innervation in diabetic and control mice. Data are presented as mean ± SEM, n = 6 mice in each group, ** *P* < 0.01, Mann-Whitney test in **(D)** and unpaired t-test in **(F)**. NMJs, neuromuscular junctions, AChRs, acetylcholine receptors; TA, tibialis anterior; CSA, cross sectional area.

Contractile activity of skeletal muscle is controlled by motor neuron axons through neuromuscular junction (NMJ), a highly specialized chemical synapse (26). The NMJ structure comprises four components: presynaptic nerve terminals, terminal Schwann cells (tSCs), synaptic cleft and postsynaptic acetylcholine receptors (AChRs). Impairments in the NMJ structure leads to muscle atrophy and declined neuromuscular action potential transmission (25, 27). The decreased CMAP amplitude observed in db/db mice indicated reduced synaptic transmission efficacy and possible structural abnormalities of the NMJ. Therefore, we evaluated NMJ structure with immunostaining, the motor axons and presynaptic terminals were labeled with NEFH and synaptophysin, and AChRs are visualized with α-bungarotoxin (α-BTX). In control mice, the NMJ was completely innervated by motor axons and the AChRs fully colocalizes with presynaptic motor nerve terminals. However, some AChRs were exposed without presynaptic nerve terminal innervation in db/db mice, suggesting that the NMJ was partially denervated (**Figures 6A, B**). In addition, the postsynaptic AChRs clusters were fine and compact, displaying a pretzel-like structure in control mice, while they became relatively more dispersed and displayed extensive fragmentation in db/db mice. The number of AChR cluster fragments significantly increased in db/db mice compared to controls (**Figures 6C, D**). Collectively, morphological analysis of the NMJ revealed structural deficits during late stages of diabetes, which probably contribute to impaired neuromuscular transmission in these animals.

Muscle spindle within skeletal muscle is the primary proprioceptive sensory organs and relays information of muscle length and movement to the spinal cord circuits (28, 29). Proprioceptive feedback from muscle spindle is critical for movement control and also essential for locomotor recovery after spinal cord injury (30–32). The sensorimotor deficits revealed by beam-walking test (**Figures 2C, D**) was hypothesized to muscle spindle abnormality, prompting us to analyze the muscle spindle morphology. H&E staining of the muscle spindle in the TA muscle showed evident atrophy of intrafusal muscle fibers in diabetic mice (**Figure 6E**). The CSA of intrafusal muscle fiber was markedly decreased in db/db mice compared with control mice (**Figure 6F**).

The equatorial region of intrafusal fibers is innervated by group Ia afferents of proprioceptive neurons that form spiral endings around the center of the intrafusal fiber, and respond to the state of muscle contraction (28, 29, 33). To determine whether muscle spindle innervation was affected in late stages of diabetes, we visualized the Ia afferents using NEFH immunostaining. In nondiabetic mice, the Ia afferents exhibit characteristic annulospiral morphology. However, the Ia afferents were irregular and disordered in db/db mice, indicating aberrant innervation of muscle spindle (**Figure 6G**). The abnormal morphology and innervation of muscle spindle might be associated with the impaired proprioception and sensorimotor behavioral dysfunction observed in db/db mice.

### 3.6 The VGLUT1-positive boutons on motor neurons were altered in db/db mice

**Figure 7:**
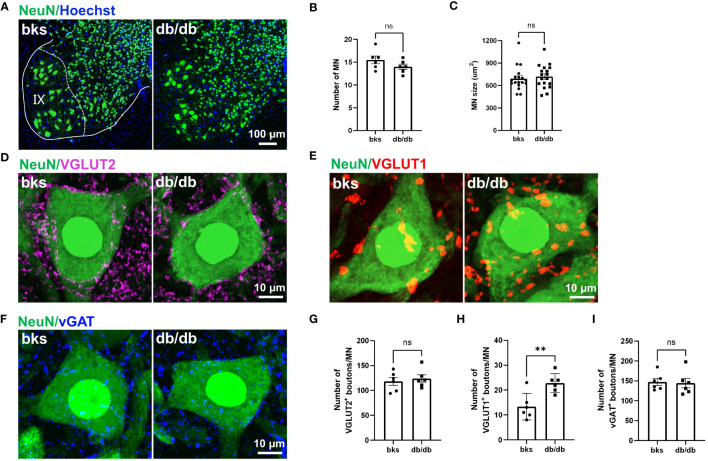
Characterization of synaptic inputs onto motor neurons in diabetic mice at 32 weeks of age. **(A)** Visualization of motor neurons in IX lamina with NeuN immunostaining. **(B, C)** Quantification of number **(B)** and size **(C)** of motor neurons in **(A)**. n = 6 mice per group and 3 motor neurons per animal were quantified in **(C)**. **(D–F)** Representative immunostaining of excitatory and inhibitory terminals projecting onto motor neurons in diabetic and control mice. VGLUT2-positive boutons **(D)**, VGLUT1-positive boutons **(E)**, and vGAT-positive boutons **(F)**. **(G–I)** Quantification of synaptic boutons **(D–F)** are shown in **(G–I)**, respectively. Data are presented as mean ± SEM, n = 6 mice in each group, **P < 0.01, ns, no significant difference, unpaired t-test in **(B, G–I)** and Mann-Whitney test in **(C)**. MN, motor neuron.

Motor neurons within the spinal ventral horn control the rhythmic contraction of skeleton muscles and maintain postural stability smooth movement at different speeds (30, 34). To determine whether the motor neurons are affected in late stages of diabetes, we detected the number and size of motor neurons within the L4-L6 spinal segments that control hindlimb movements. We marked neurons with NeuN immunostaining, and NeuN-positive neurons with large cell bodies (>360 μm^2^) that located in lamina IX were identified as motor neurons (**Figure 7A**). The number of motor neurons was similar between the diabetic and control mice (**Figure 7B**). In addition, the size and morphology of motor neurons were not changed either in db/db mice in comparison to controls as indicated in **Figure 7C**, suggesting that motor neurons are relatively more resistant during diabetes progression.

Motor neurons within the ventral spinal cord integrate diverse synaptic inputs from the supraspinal descending pathways, spinal interneurons, and peripheral sensory feedback to generate the rhythm and pattern of locomotion and mediate the whole-body movement (35, 36). These inputs are mainly divided into two categories: excitatory inputs from glutamatergic neurons and inhibitory inputs from GABAergic or glycinergic neurons. Although motor neuron loss and morphological alteration have not been observed at late stages of diabetes, it remains unclear whether the synaptic inputs onto motor neurons are affected. In the following work, we marked glutamatergic terminals with VGLUT1 and VGLUT2 immunostaining to visualize the excitatory synaptic inputs onto motor neurons. Abundant VGLUT2-positive terminals were found on motor neuron somata in diabetic and control mice. The number of VGLUT2-positive contacts on motoneurons was similar between the two groups (**Figures 7D, G**). Interestingly, we observed a profound increase in the number of VGLUT1-positive boutons contacting motoneuron somata in db/db mice in comparison to controls (**Figures 7E, H**). The VGLUT1-positive terminals in the spinal cord are mostly derived from the proprioceptive afferent neurons in the DRG. Therefore, the increased VGLUT1-positive contacts may indicate elevated projections of proprioceptive afferents onto motor neurons. We then labeled the inhibitory inputs onto motor neurons with vGAT immunostaining, and found that there was no significant change in the number of vGAT-positive inhibitory boutons on motor neurons between the two groups (**Figures 7F, I**). Taken together, these findings suggested that VGLUT1-positive terminals were more susceptible to diabetic neuropathy, and this imbalance in excitatory-inhibitory synaptic inputs to motor neurons might disturb locomotor rhythm generation and account for the impaired limb coordination observed in db/db mice.

## 4 Discussion

Diabetic neuropathy is the most prevalent diabetes-related complication, leading to sensory, motor and autonomic dysfunction, reduced quality of life, and increased mortality in patients with type 1 or type 2 diabetes (2). In the present study, we used the obese db/db diabetic mouse model to test sensory and motor abnormalities and the related morphological changes in the nervous system in late stages of type 2 diabetes.

Diabetic neuropathy has been widely studied at the early stages of the disease, and we further confirmed that db/db mice displayed hyperglycemia and obesity persistent during late-stage diabetes accompanied by substantial loss of insulin-producing β-cells in pancreas. At later time points of diabetes, the db/db mice displayed evident sensory neuropathy confirmed by decreased hindpaw sensitivity to mechanical and thermal stimuli compared with age-matched controls. The impaired sensory response was largely attributed to extensive epidermal denervation in the footpads of diabetic mice as diabetes progressed. Immunostaining showed remarkable loss of PGP9.5-positive IENFs, particularly of CGRP-positive nociceptive fibers in db/db mice. However, it remains unclear whether the central terminals of primary afferent neurons also undergone degeneration.

We then examined the central distribution of CGRP-positive peptidergic fibers, IB4-positive nonpeptidergic fibers, and NEFH-positive myelinated fibers in the SDH. Unexpectedly, the distribution of CGRP-positive fibers was upregulated in db/db mice in comparison to controls, the CGRP-positive fibers extended from the superficial laminae (I-II) to the deep laminae (III-IV). However, the area of IB4-positive fibers and NEFH-positive fibers was similar between the two groups. CGRP-positive peptidergic neurons are required to sense noxious stimuli and have key roles in the development and maintenance of neuropathic pain (37, 38). Previous studies have shown that upregulation of CGRP-positive fiber in the dorsal horn of spinal cord is the pathological basis of long-term hyperalgesia. CGRP-positive terminal sprouting and redistribution in the SDH have been reported in a variety of pain hypersensitivity conditions, such as neuropathic pain induced by anticancer agents (39), sciatic nerve compression, and spinal cord injury (21, 40). However, few studies have tested the distribution and expression of CGRP-positive terminals in the SDH during diabetic neuropathy. Although distal symmetrical polyneuropathy with numbness in the distal extremities is the common presentation of diabetic neuropathy (41), approximately 25-30% of patients with diabetes experience diabetic neuropathic pain (DNP) (6). The management of DNP is challenging and lacks effective treatments because the mechanisms underlying DNP are complicated and not completely elucidated. The sprouting and redistribution of CGRP-positive terminals found in our study might serve as the synaptic and structural basis for chronic pain, and provide a new therapeutic target for diabetic neuropathic pain. It has been reported that microglia activation was involved in sprouting of CGRP-positive fibers in chronic pain, and microglia activation was also observed in our db/db mice. The activation of microglia might participate in the sprouting of CGRP-positive terminals. Further studies are needed to confirm this phenomenon and to elucidate the underlying mechanisms potentially linking microglial activation and CGRP-positive terminal sprouting during late-stage diabetes.

The db/db mice also displayed motor dysfunction in the rotarod test and beam-walking test at late stages of diabetes. We next detected morphological alterations in neural circuits controlling movement. We found that diabetes induced severe muscle atrophy of lower limbs, which was further confirmed by histological analysis in CSA of muscle fibers. Generally, diabetic muscular atrophy is considered as a result of microvasculature dysfunction and demyelination of peripheral nerves (42). However, several studies have demonstrated deficient neuromuscular transmission, innervation and morphological structure in several murine models of diabetes during diabetes neuropathy (43–47). Similar to previous studies, we found that db/db mice exhibited deficits in neuromuscular transmission as revealed by decreased CMAP amplitude. Nerve impulses from spinal motor neurons are transmitted to muscle fibers via the NMJ in a 1:1 ration in vertebrates (25). Impairment in NMJ formation and maintenance leads to neuromuscular disorders, muscle atrophy or weakness (48). We speculated that the downregulated CMAP amplitude might be a hint of NMJ dysfunction apart from loss of myelinated motor axons, and the subsequent morphological analysis of NMJs confirmed our hypothesis. Specifically, the NMJs of diabetic mice were partially denervated with fragmentation of AChR clusters. The tSCs play a crucial role in NMJ formation, maintenance, regeneration, and remodeling (49–51), and an interesting topic for future studies is whether loss of tSCs contribute to diabetes-induced NMJ deficits. Additionally, the number of pre-synaptic active zones and the depth of postsynaptic folds are key factors determining the synaptic efficacy of NMJs. Further transmission electron microscopy analysis is necessary to determine whether these structures are altered and involved in NMJ deficits in db/db mice.

To confirm whether motor neurons were affected in db/db mice, we firstly visualized motor neurons by NeuN staining and calculated the number and size of motor neurons. Consistent with our assumption, diabetes did not affect the quantity and morphological properties of motor neurons even in late diabetes progression. This result differs from a previous report, which demonstrated a significant decrease in the number and size of GM motor neurons in a rat model of diabetes (52). This discrepancy may due to the different animal models used in the study (the db/db mouse model of type 2 diabetes in our study versus a STZ-induced rat model of type 1 diabetes in the previous study). The pathological change probably varies in the spinal cord between two distinct models. Furthermore, the detection technique was different. The previous study adopted retrograde labeling technique to detect GM motor neurons, however, retrograde labelling may not thoroughly visualize motor neurons since the retrograde axonal transport is reduced in the context of diabetes (52). On the contrary, we labeled the overall alpha motor neurons by NeuN immunostaining, which is relatively more comprehensive. It should be noted that there are two types of motor neurons innervating different muscle targets in the spinal cord: Alpha motor neurons predominately innervate extrafusal muscle fibers, and gamma motor neurons innervate the intrafusal muscle fibers. Alpha motor neurons have a high-level of NeuN expression, whereas gamma motor neurons lack expression of NeuN (53). Therefore, the NeuN immunostaining in our study did not detect gamma motor neurons. Whether and how diabetes affects spinal gamma motor neurons that modulating muscle spindle remains unclear and should be further investigated in the future.

A previous study demonstrated that STZ-induced diabetic mice aged 10 weeks displayed significant sensorimotor dysfunction using the beam-walking apparatus (54). Consistent with their findings, the db/db mice aged 32 weeks in our study exhibited severe sensorimotor behavioral deficits. The db/db mice had more foot slips and took longer time when crossing the beam-walking apparatus compared with controls. We found that the db/db mice displayed intrafusal muscle fiber atrophy and aberrant muscle spindle innervation, which probably contributed to their sensorimotor behavioral deficits by reducing the sensitivity of muscle spindle to muscle contraction. Group Ia proprioceptive afferents innervating muscle spindle form VGLUT1-positive excitatory monosynaptic connections with α-motor neurons to refine motor output (29, 55). We found that the VGLUT1-positive boutons contacting motor neurons were markedly upregulated in db/db mice, while VGLUT2-positive and vGAT-positive punctae on motor neurons were not altered. In the spinal cord, the vast majority of VGLUT1-positive terminals onto motor neurons is derived from proprioceptive Ia afferents, and another small fraction (2%) derives from CST terminals (56). We hypothesized that the increased number of VGLUT1-positive synaptic boutons might be due to terminal sprouting of proprioceptive Ia afferents. The mechanisms underlying this sprouting remains unclear, and two possible mechanisms were considered for this sprouting. Firstly, sprouting may be a compensatory response to the disorganized muscle spindle innervation. The disordered Ia innervation of muscle spindle may lead to altered Ia firing patterns and subsequently affected excitatory drive to motor neurons. In this scenario, the Ia afferents increased terminal sprouting to enhance proprioceptive inputs and provided sufficient excitatory drive to match the movement. Secondly, previous studies have demonstrated that the cytokines and growth factors released from activated microglia contributed to synaptic structural plasticity of local nerve fibers (57, 58). Our findings are consistent with the possibility that diabetes-induced microglial activation might promote sprouting of proprioceptive Ia afferents, but further studies are needed to thoroughly test this hypothesis.

In summary, we document severe motor dysfunction apart from sensory neuropathy in late stages of type 2 diabetes in a validated mouse model. These findings expand our understanding of the functional and morphological changes affecting motor and sensory systems in diabetes. Future studies are needed to explore the mechanisms underlying the histological alterations and to identify novel strategies to alleviate the behavioral deficits during late type 2 diabetes.

## Data availability statement

The original contributions presented in the study are included in the article/supplementary material. Further inquiries can be directed to the corresponding authors.

## Ethics statement

The animal study was approved by Institutional Animal Care and Use Committee (IACUC) of Laboratory Animal Resources Center, Tsinghua University. The study was conducted in accordance with the local legislation and institutional requirements.

## Author contributions

TT: Writing – review & editing, Writing – original draft. HL: Writing – review & editing, Methodology. SZ: Writing – review & editing, Methodology, Investigation. MY: Writing – review & editing, Supervision, Funding acquisition, Conceptualization.
